# Implementing Arithmetic and Other Analytic Operations By Transcriptional Regulation

**DOI:** 10.1371/journal.pcbi.1000064

**Published:** 2008-05-09

**Authors:** Sean M. Cory, Theodore J. Perkins

**Affiliations:** 1 Department of Human Genetics, McGill University, Montreal, Quebec, Canada; 2 School of Computer Science, McGill University, McGill Centre for Bioinformatics, Montreal, Quebec, Canada; University of Illinois Urbana-Champaign, United States of America

## Abstract

The transcriptional regulatory machinery of a gene can be viewed as a computational device, with transcription factor concentrations as inputs and expression level as the output. This view begs the question: what kinds of computations are possible? We show that different parameterizations of a simple chemical kinetic model of transcriptional regulation are able to approximate all four standard arithmetic operations: addition, subtraction, multiplication, and division, as well as various equality and inequality operations. This contrasts with other studies that emphasize logical or digital notions of computation in biological networks. We analyze the accuracy and precision of these approximations, showing that they depend on different sets of parameters, and are thus independently tunable. We demonstrate that networks of these “arithmetic” genes can be combined to accomplish yet more complicated computations by designing and simulating a network that detects statistically significant elevations in a time-varying signal. We also consider the much more general problem of approximating analytic functions, showing that this can be achieved by allowing multiple transcription factor binding sites on the promoter. These observations are important for the interpretation of naturally occurring networks and imply new possibilities for the design of synthetic networks.

## Author Summary

The biochemistry of the cell is daunting in its complexity. In order to understand this complexity, we are often forced to use metaphors or construct analogies to systems that we understand better. One long-standing analogy is to digital computers, with their large networks of interacting components that manage to act in coherent and useful ways. Indeed, we know from both theoretical models and empirical observations that biological entities such as genes can sometimes be described accurately by digital, or logical, expressions—turning on or off in response to regulatory signals. However, far more sophisticated computations can also take place, as has been documented in the responses of genes such as the *lac* operon or Endo-16. We analyze chemical kinetic models of transcriptional gene regulation and show that even simple models are capable of nearly arbitrary analog computations, ranging from elementary arithmetic operations to general analytic functions. Understanding the computational capacities of genes, and of biochemical systems more generally, tells us what to look for when studying natural systems and tells us what we can hope to build by biological engineering.

## Introduction

In grappling with the biochemical complexity of gene regulation, some have turned to computational metaphors for explaining gene behavior (e.g., [1]–[5]). The *lac* operon, for example, is often described as implementing a simple logical rule—the gene is on when lactose is present and glucose is absent [6]. Theoretically, a variety of nonlinear chemical systems, including gene regulatory systems, are capable of implementing arbitrary logical rules [7]–[11]. Networks of such systems can implement finite state machines [12], and families of such networks of increasing size can be said to implement arbitrary Turing-computable functions [13],[14]. In practice, logical models have proven capable of accounting for the qualitative dynamics of a variety of genetic systems [15]–[23]. Models that combine logical rules with concentration thresholds for the action of regulatory molecules, as in the French-flag model of Wolpert [24] or Glass networks more generally [25], satisfactorily describe other systems either qualitatively [26]–[28] or quantitatively [29]. Synthetic biologists have constructed gene networks that perform elementary logical operations, such as storing a bit of memory [30] or turning off and on with a fixed period [31].

However, detailed analysis of transcriptional regulatory networks reveals a behavior richer than logical responses. Yuh *et al.*'s model of the sea urchin developmental gene Endo-16 contains logical as well as additive and multiplicative operations [32]. Recent measurements of *lac* transcription rate as a function of the concentrations of cAMP and an analogue of allolactose show four plateaus of different rates connected by smooth boundaries [4],[5]. Even “logical” models of gene regulation often require more than two qualitatively distinct levels of gene expression (e.g., [19]), recognizing that some genes cannot be treated simply as on or off.

Besides logical or digital computation, several other notions of computation have been explored. Analog computations made by artificial neural networks can in principle be implemented by chemical systems [8],[12],[33]. Deckhard and Sauro experimented with evolving reaction networks to compute square and cubic roots of an input [34] and have since evolved networks to compute a variety of other arithmetic functions. This work shares the greatest commonality withours, the differences being that we specifically study single-gene transcriptional regulatory networks and that our designs are arrived at prescriptively, by analysis of the steady state equations, rather than by a computational search.

A different line of reasoning has focussed on the robustness of biochemical networks to variability in inputs and parameters—intuitively important features for real systems [35]–[37]. Reduction in noise by development gene networks was experimentally observed [38],[39] and confirmed as a property of mathematical models [20],[40]. In this background, control theoretic concepts, especially noise filtering, were studied more carefully [41] and found in a wide variety of systems (see [37] for a summary). More recently, the behavior of several genes has been viewed in the context of information theory [42] and Bayesian decision theory [43].

The present work focuses on the general steady-state analog computational capacities of genes. Most of the paper considers a simple chemical kinetic model of transcriptional regulation for a single gene with two transcription factors. We assume that concentrations of proteins represent non-negative real numbers, with the transcription factor concentrations acting as inputs to the gene and the steady state expression of the gene acting as the output. Different choices for the kinetic rates allow the gene to approximate different binary arithmetic operations: addition, subtraction, multiplication, division, and testing for equality and various inequalities. Variations on the model allow alternative implementations of the same functions as well as other functions, such as the square root. In the model, these operations can be reproduced with arbitrary accuracy, although in reality, biological limits on the parameters would limit accuracy. We analyze the accuracy of these approximations in terms of the deviation between the steady state output and the desired output. We also analyze the precision of the approximations, in terms of the variability of the output over time in a stochastic interpretation of the model. Through theoretical analysis and simulations we show that such “arithmetic genes” can be combined in networks to compute more sophisticated functions. As an example, we describe an eight gene network that tracks the mean and standard deviation of a time-varying signal and detects times at which the signal is statistically significantly elevated. We also consider a model of a gene regulated by a single type of transcription factor but having multiple binding sites on the promoter. With such a gene, arbitrary analytic functions can be approximated up to a fixed order based on power series expansions. We demonstrate by designing a gene that approximates the cosine function.

## Results

### A chemical-kinetic model of transcriptional regulation

**Figure 1:**
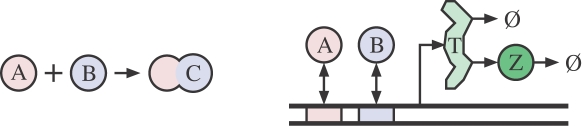
Schematic of a chemical model of a gene regulated by two transcription factors. Transcription factors *A* and *B* may irreversibly form an inert dimer, *C*, or they may bind individually or simultaneously to the promoter region of the gene, where they affect the transcription rate. Transcripts, *T*, are translated into proteins, *Z*. Both *T* and *Z* decay at fixed rates.

Figure 1 presents a diagram of the model of transcriptional regulation and gene expression that we analyze. It models a single gene regulated by two transcription factors, *A* and *B*. These factors may bind irreversibly to form an inertdimer, or they may bind to the DNA, where they affect the rate of transcription. Transcripts are translated into proteins at a fixed rate, and both transcripts and proteins decay at fixed rates. The reaction equations below formalize the model. (Please note that Tables 1, 2, and 3 show the symbols employed in this paper and their meanings.)
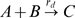
(1)

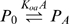
(2)

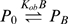
(3)

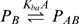
(4)

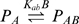
(5)

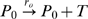
(6)

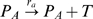
(7)

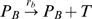
(8)

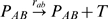
(9)

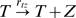
(10)

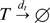
(11)

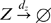
(12)For the bidirectional reactions, equilibrium association constants are ratios of forward to backward rates (e.g., *K* *_oa_* = *f_oa_*/*b_oa_*). We use [*X*] to denote the steady state concentration of molecular species *X*. None of the reactions create or destroy the factors *A* or *B*. We assume that the binding of single molecules of these factors to the DNA does not significantly affect the concentration of free *A* or *B*, so that [*A*]+[*C*] = [*A_tot_*] and [*B*]+[*C*] = [*B_tot_*], where [*A_tot_*] and [*B_tot_*] denote the total concentration of molecules *A* and *B* respectively, either bound or unbound.

**Table 1:** Symbols that pertain throughout the paper and that are used in particular for the the description and analysis of arithmetic genes.

Symbol	Meaning
*A*	Transcription factor *A*
*B*	Transcription factor *B*
*C*	Inert heterodimer of *A* and *B*
*T*	Transcript
*Z*	Protein
*P*_0_	Promoter complex unbound
*P_A_*	Promoter complex bound by *A*
*P_B_*	Promoter complex bound by *B*
*P_AB_*	Promoter complex bound by *A* and *B*
*r_d_*	Rate of dimerization of *A* and *B*
*K_oa_*	Association constant for *A* binding to promoter
*K_ob_*	Association constant for *B* binding to promoter
*K_ab_*	Association constant for *B* binding to promoter after *A*
*K_ba_*	Association constant for *A* binding to promoter after *B*
*f_ij_*	Forward (binding) rate for one of the above association constants
*b_ij_*	Backward (unbinding) rate for one of the above association constants
*r_o_*	Transcription rate when promoter unbound
*r_a_*	Transcription rate when promoter bound by *A*
*r_b_*	Transcription rate when promoter bound by *B*
*r_ab_*	Transcription rate when promoter bound by *A* and *B*
*r_t_*	Net rate of transcription
*r_tz_*	Translation rate
*d_t_*	Transcript decay rate
*d_z_*	Protein decay rate
[*X*]	Steady state concentration of species *X*
*X_tot_*	Total amount of species *X* in the system
*X*(*t*)	Concentration of species *X* at time *t*
τ*_o_*	Fraction of time promoter unbound
τ*_a_*	Fraction of time promoter bound by *A*
τ*_b_*	Fraction of time promoter bound by *B*
τ*_ab_*	Fraction of time promoter bound by *A* and *B*
*Z_on_*	Target “on” concentration for *Z* for comparison operations
*r_z_*	Dimerization rate of protein *Z*, for the square root function
*Z*_2_	Homodimer of protein *Z*
ρ	Scale factor for *A* and *B* binding and unbinding rates used in noise simulations

**Table 2:** Symbols that are used in the context of the network for detecting significant elevation in a time-varying signal.

Symbol	Meaning
*I*	Input signal
μ*_I_*	Mean of *I* over time
σ*_I_*	Standard deviation of *I* over time
*c*	Number of standard deviations above mean deemed significantly elevated
*T^i^*	Concentration of transcripts for gene G*i*
*Z^i^*	Concentration of protein for gene G*i*

**Table 3:** Symbols that are used in the context of the gene for approximating analytic functions.

Symbol	Meaning
*N*	Number of binding sites for *A* on promoter
*P_i_*	Promoter bound by *i* copies of *A*
*K*	Association constant for *A* binding to promoter
*r_i_*	Transcription rate when promoter bound by *i* copies of *A*

Most of our analysis concerns the steady state behavior of this system. We use τ*_o_*, τ*_a_*, τ*_b_*, and τ*_ab_* to denote the fractions of time that the promoter spends unbound or bound by different combinations of transcription factors. We assume that the reactions for transcription factors binding to the promoter are at equilibrium.
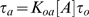
(13)

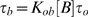
(14)

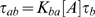
(15)

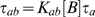
(16)From this system of equations, one can deduce that *K_oa_K_ab_* = *K_ob_K_ba_*. However, the system is degenerate and does not lead to a solution for the occupancy times until we recognize that the occupancy times must sum to one.
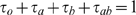
(17)With the addition of this equation, we obtain four linearly independent equations, which can be manipulated to solve for the occupancy times.
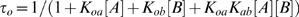
(18)

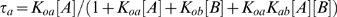
(19)

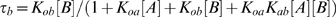
(20)

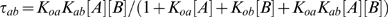
(21)This allows us to calculate the net rate of transcription.
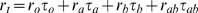
(22)The steady state concentration of protein *Z* then follows.
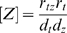
(23)

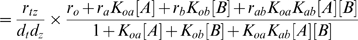
(24)Because *K_oa_K_ab_* = *K_ob_K_ba_*, the term *K_oa_K_ab_* can be replaced by *K_ob_K_ba_* in any of the equations above. These equations are also true for more restricted binding scenarios, including: *A* and *B* cannot be bound to the DNA simultaneously (signified by *K_ab_* = *K_ba_* = 0); *A* must bind before *B* binds, and *B* must unbind before *A* can unbind (*K_ob_* = *K_ba_* = 0); and *B* cannot bind the DNA at all (*K_ob_* = *K_ba_* = *K_ab_* = 0). In these alternative scenarios, however, it no longer holds that *K_oa_K_ab_* = *K_ob_K_ba_*.

### Approximating arithmetic operations

**Figure 2:**
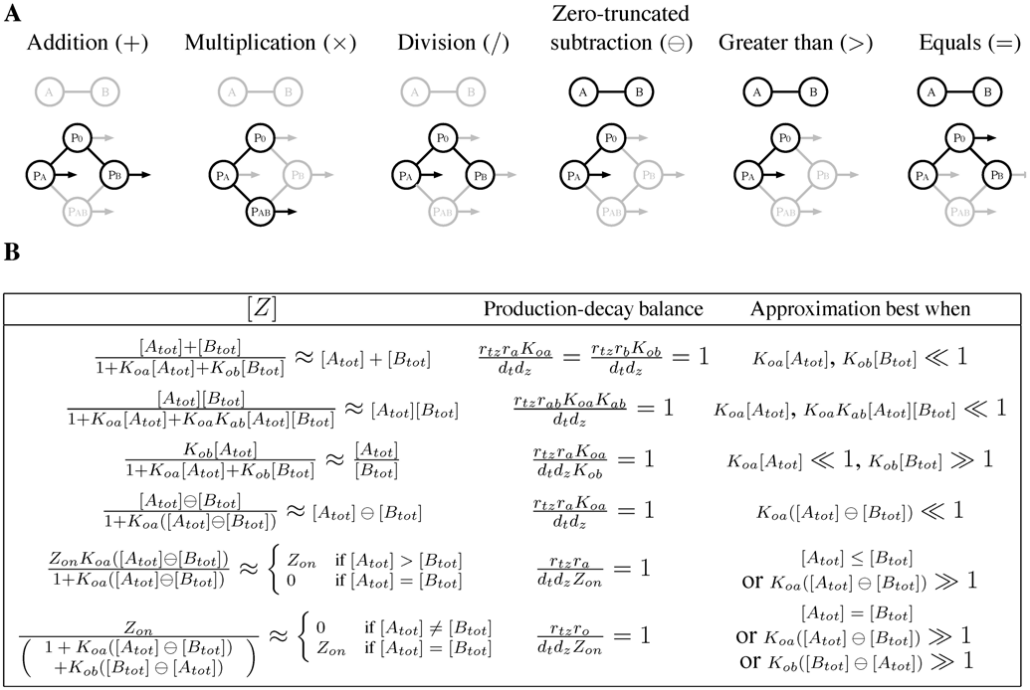
Regulatory architectures and parameters for approximating various arithmetic and comparison operations. (A) Diagrams depict the reactions employed to achieve each operation. The *A*–*B* dumbbell is bold if *A* and *B* dimerize and gray if they do not. The four circles connected as a diamond represent different binding states of the promoter. In bold are achievable binding states, with bold connecting bars indicating the allowed transitions. A bold arrow leaving a circle to the right indicates a binding state in which transcription occurs. (B) Steady state expression and parameter constraints. Each row of the table corresponds to one operation. The [*Z*] column gives the exact and approximate steady-state expression of the gene. The exact steady state is obtained from Equation (24), assuming parameters conform to the formulae in the “Production-decay balance” column and setting to zero those parameters implied to be zero by the diagrams in (A). The final column of the table describes under what conditions each operation is well approximated. The symbol ⊝ denotes zero-truncated subtraction, defined as *x*⊝*y* = max(*x*–*y*, 0).

By employing subsets of the allowed reactions and setting kinetic parameters appropriately, different arithmetic operations can be approximated, with [*A_tot_*] and [*B_tot_*] treated as the inputs. (See Figure 2 for a summary.) For example, consider Equation 24 under the conditions: (i) *r_d_* = 0, (ii) *K_ab_* = *K_ba_* = 0, (iii) *r_o_* = 0, (iv) *r_tz_r_a_K_oa_* = *r_tz_r_b_K_ob_* = *d_t_d_z_*, (v) *K_oa_*[*A*]≪1, and (vi) *K_ob_*[*B*]≪1. These conditions can be interpreted as: (i) *A* and *B* do not dimerize, (ii) *A* and *B* cannot both be bound to the DNA at the same time, perhaps because their binding sites overlap, (iii) there is not transcription if neither *A* nor *B* are bound to the DNA, (iv) there is a certain balance between the production and decay rates of mRNA and protein, and (v),(vi) either *A* and *B* bind to the DNA comparatively weakly or else we are considering only comparatively small concentrations of *A* and *B*. Then Equation 24 tells us that [*Z*]≈[*A_tot_*]+[*B_tot_*], so that the gene approximates the addition operation. (See Figure 2B for the exact steady state expression.) The error in the approximation is analyzed in more detail in the next section.

Alternatively, suppose that *A* and *B* bind the promoter sequentially, perhaps because *A* is a cofactor without which *B* cannot bind, and that transcription is activated only when both are bound. If production and decay are balanced as *r_tz_r_ab_K_oa_K_ab_* = *d_t_d_z_* and if *K_oa_*[*A_tot_*]≪1 and *K_oa_K_ab_*[*A_tot_*][*B_tot_*]≪1, then [*Z*]≈[*A_tot_*][*B_tot_*] so that the gene approximates the multiplication operation. To achieve [*Z*]≈[*A_tot_*]/[*B_tot_*] one can assume that *A* and *B* can bind individually to promoter, and that *A* activates transcription whereas *B* represses. This approximation is most accurate when *B* binds strongly, so that *K_ob_*[*B_tot_*]≫1, and *A* binds comparatively weakly, so that *K_oa_*[*A_tot_*]≪*K_ob_*[*B_tot_*].

As [*Z*] is always non-negative, one cannot approximate ordinary subtraction, [*A_tot_*]−[*B_tot_*], when [*B_tot_*]>[*A_tot_*]. Instead, we consider zero-truncated subtraction.
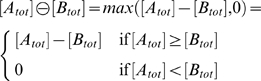
(25)If *A* and *B* dimerize irreversibly with forward rate *r_d_*>0, then at steady state *C* = min([*A_tot_*],[*B_tot_*]). Thus, [*A*] = [*A_tot_*]−[*C*] = [*A_tot_*]−min([*A_tot_*],[*B_tot_*]) = [*A_tot_*]⊖[*B_tot_*]. Dimerization itself, then, solves the zero-truncated subtraction problem. If we desire [*Z*]≈[*A_tot_*]⊖[*B_tot_*], we need only assume that undimerized *A* binds to the promoter and activates transcription. For an accurate approximation, binding should be weak, so that *K_oa_*[*A*]≪1.

For the comparison operations, the goal is [*Z*] = 0 if the comparison is false and [*Z*] = *Z_on_* if the comparison is true, for some chosen *Z_on_*>0. The operation [*A_tot_*]>[*B_tot_*] can be implemented with the same set of reactions as subtraction, but *A* should bind the promoter strongly so that [*Z*]≈*Z_on_* in the presence of any amount of undimerized *A*. For [*A_tot_*] = [*B_tot_*] we assume that *A* and *B* dimerize, that *A* and *B* can individually bind the promoter, acting as repressors. If [*A_tot_*] = [*B_tot_*], then there will be no undimerized *A* or *B*, and [*Z*] = *Z_on_*. Otherwise, there will be some undimerized *A* or *B*, and, if it binds the promoter strongly, will turn off transcription so that [*Z*]≈0.

These arithmetic operations can be implemented in other ways, even restricting attention to the model in Figure 1. For example, the addition gene could allow *A* and *B* to bind simultaneously, as long as there is no transcription when both are bound. Similarly, the multiplication gene could allow *A* and *B* to bind to the promoter in either order, as long as there is only transcription when both are bound. However, these alternate versions are less accurate than the designs in Figure reffig∶diagrams because they introduce unnecessary promoter binding states. Mathematically, the decrease in accuracy corresponds to extra terms appearing in the denominator of the second term in Equation 24. Variations on these models can produce other useful functions. Trivial modifications allow the other comparison operations: greater-than-or-equal, less-than, and less-than-or-equal. More interestingly, consider a model with a single transcription factor *A*, which can be obtained by dropping all equations from the model that involve *B*. Suppose that *A* activates transcription. Further, suppose that *Z* dimerizes at forward rate *r_z_* and that the dimer itself is inert and degrades at some rate *d_z_*, but that *Z* on its own does not decay. Then Equation 12 is replaced by
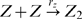
(26)

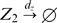
(27)If *r_tz_K_oa_r_a_* = *d_t_r_z_* and *K_oa_*[*A_tot_*]≪1, then at steady state 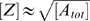. Genes that approximate other fractional powers can be constructed by assuming more complicated promoter-binding or degradation schemes.

### Accuracy depends on promoter occupancy

**Figure 3:**
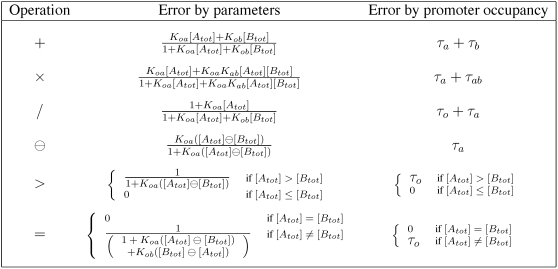
Accuracy with which the genetic designs in Figure 2 approximate the intended operations. We quantify accuracy in terms of relative error—|*f*([*A_tot_*],[*B_tot_*])−[*Z*]|/*f*([*A_tot_*],[*B_tot_*]) for the arithmetic operations, and |*f*([*A_tot_*],[*B_tot_*])−[*Z*]|/*Z_on_* for the comparison operations, where *f* is the operation being approximated. The second column in the table gives relative error in terms of the kinetic parameters. The third column gives the relative error in terms of the fraction of time the promoter spends bound by different combinations of transcription factors at steady state.

The accuracy with which the models in Figure 2 approximate the desired operations depends on the kinetic parameters as well as the transcription factor concentrations [*A_tot_*] and [*B_tot_*]. We analyze accuracy in terms of relative error. For the arithmetic operations of addition, multiplication, division, and zero-truncated subtraction, we define relative error as 
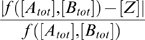
, where *f* is the desired operation. This is not well defined when *f* is zero. However, for the models in Figure 2, [*Z*] = 0 whenever *f* is zero, so we can take the relative error to be zero. For the comparison operations we define relative error as 
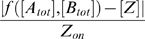
. The relative error can be calculated by substituting the formula for [*Z*] in Figure 2B into the definition for relative error. Figure 3 shows the relative errors of the six gene models from Figure 2. An advantage to studying this notion of accuracy is that the relative error can be easily expressed in terms of the fraction of time the promoter spends in different binding states (Figure 3, third column).

For the arithmetic operations, relative error is generally increasing in [*A_tot_*] and [*B_tot_*] as well as the equilibrium association constants for the binding of *A* and *B* to the DNA (Figure 3, second column). Thus, the approximations are most accurate when the concentrations of the transcription factors are small or when they bind weakly to the DNA—as already emphasized in the third column of Figure 2B. Intuitively, this keeps the transcriptional response in the linear regime. Saturation of the transcription rate, due to high concentration of transcription factor(s) or strong transcription factor binding, reduces accuracy. Indeed, the relative error of the addition, multiplication and zero-truncated subtraction genes is simply the fraction of time that the DNA is bound by either transcription factor. The division gene is a partial exception to these rules. First, its relative error is decreasing in *K_ob_* and [*B_tot_*], not increasing. Second, the relative error is equal to the fraction of time the DNA is not bound by *B*.

The effects of these parameters cannot be considered in isolation, however, because the parameters as a group must satisfy the production-decay balance shown in Figure 2B. For the addition gene, for example, small transcription factor association constants, which result in an accurate approximation, must be balanced by large rates of transcription and/or translation or small rates of transcript and/or protein decay.

For the comparison operations, accuracy is highest when *A* and *B* bind strongly to the promoter. If *K_oa_* and *K_ob_* are small and if [*A_tot_*] and [*B_tot_*] are just slightly different, then there is only a small amount of undimerized transcription factor binding weakly to the promoter, and this does not provide sufficient transcriptional activation (in the case of >) or repression (in the case of  = ). For either gene, the relative error is either zero or τ*_o_*, depending on whether the comparison is true or false.

### Noise in the output is independent of accuracy

In a stochastic kinetics interpretation of the model in Figure 1 (Equations 1 to 12), the output protein concentration varies over time even if [*A_tot_*] and [*B_tot_*] are fixed. Let *X*(*t*) denote the concentration of molecular species *X* at time *t*. Noise in *Z*(*t*), defined as the standard deviation of *Z*(*t*) over time divided by the mean of *Z*(*t*) over time, can be attributed to three sources: (i) inherent fluctuations due to the stochastic birth-death process for *Z*, (ii) variability in *T*(*t*) due to its own inherently stochastic birth-death process, which in turn creates variability in the production rate of *Z*, and (iii) variability in the promoter state, which affects the production rate of *T*, and by extension, of *Z*. If one assumes that the transcription factor binding reactions are at steady state, then the third source of noise is absent, and the noise in *Z*(*t*) is equivalent to that in the simpler system
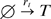
(28)

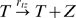
(29)

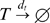
(30)

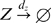
(31)where *r_t_* is the net rate of transcript production.
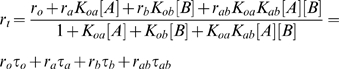
(32)For this system, the moments of *Z* can be calculated exactly from the chemical master equation, and the noise is [44]

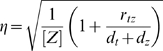
(33)Importantly, the noise in *Z*(*t*) bears no necessary relationship with the accuracy with which [*Z*] approximates a desired function, because accuracy and noise depend on different sets of parameters. For the addition gene, for example, accuracy is determined by *K_oa_* and *K_ob_*. These could be large or small regardless of the noise level, which is determined by *r_tz_*, *d_t_* and *d_z_*. This is true even considering the production-decay balance constraint for the gene, *r_tz_r_a_K_oa_* = *r_tz_r_b_K_ob_* = *d_t_d_z_*. The parameters *r_a_* and *r_b_* do not occur in the formulas for either accuracy or noise and can be used to ensure that the constraints are satisfied. This is essentially the same as the observation by Thattai and van Oudenaarden that the mean and variance of gene expression levels are controlled by independent sets of parameters [44].

**Figure 4:**
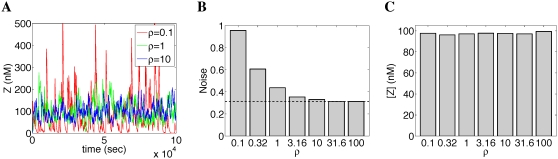
Stochastic kinetics simulation of an Addition gene, for varying rates of transcription factor binding and unbinding. Larger values of ρ correspond to both faster binding and unbinding, with no change in the equilibrium association constant. (A) Sample traces of the output, *Z*(*t*). (B) Empirical noise (standard deviation divided by mean) in the output. Dashed line gives the expected noise under the steady state assumption for the promoter. (C) Mean output, which is independent of ρ. See Materials and Methods for details, including kinetic parameters.

If the transcription factor binding reactions are not at steady state, then the third source of noise in *Z*(*t*) returns. Intuitively, however, the faster the binding and unbinding reactions are compared to the rate of transcription, the more this noise is filtered out by the slower transcription process. For example, consider the addition gene with inputs [*A_tot_*] = 30 molecules and [*B_tot_*] = 70 molecules. We used the Gillespie algorithm [45] to simulate the stochastic chemical kinetics defined by Equations 1 to 12, but replacing the transcription factor-DNA binding and unbinding rates (*f_oa_*, *f_ob_*, *b_oa_*, *b_ob_*) by scaled versions (ρ*f_oa_*, ρ*f_ob_*, ρ*b_oa_*, ρ*b_ob_*). By varying ρ, we could change the rates of binding and unbinding, while leaving *K_oa_* and *K_ob_* constant. (See Materials and Methods for the full set of kinetic parameters.) Figure 4A shows three sample traces of *Z*(*t*) for three different choices of ρ, in which the noise in *Z*(*t*) can be seen to decrease for increasing ρ. Figure 4B shows the noise in the simulated *Z*(*t*) for a wider range of ρ. For sufficiently large ρ, the steady state approximation for the promoter is good, and the noise is seen to converge to the value predicted by Equation 33, indicated by the dashed line. Figure 4C shows that the empirical mean concentration [*Z*] is at nearly the correctly value of 100, and is independent of ρ. Independence of accuracy from promoter state fluctuations has been shown analytically via Master equation analysis for some binding scenarios [46], but should hold in general for our models, as noise depends on both the binding and unbinding rates of transcription factors, whereas accuracy depends only on the ratios of the rates.

### Example: A network for detecting statistically significant elevation in a time varying signal

Arithmetic genes can be combined into networks to perform more sophisticated computations. As an example, consider a cell in which the concentration of a molecule *I* varies with time. Suppose it is important for the cell to detect times at which *I*(*t*) is significantly elevated. For example, the cell might be a bacterium and *I*(*t*) might be correlated to the concentration of an extracellular sugar. If the bacterium encounters a high-sugar environment, it may want to begin expressing the genes needed to transport and metabolize the sugar. Or, *I* may be a toxin, and high toxin levels might trigger a defensive or developmental decision, such as sporulation, to protect the bacterium. In a synthetic biology context, *I* might be a signal sent by the experimenter to trigger a response or a marker for a diseased cell that should be destroyed.

A single threshold-activated gene, for example the “greater than” gene of Figure 2, provides a simple solution to this problem. However, such a gene requires a predefined notion of how large a signal is considered elevated. Further, a chance fluctuation in the signal or in the transcriptional machinery itself might trigger a false response. The feedforward motif [47] is one way to reduce incorrect responses due to fluctuations. This motif describes a set of three genes in which gene 1 regulates gene 2, and both genes 1 and 2 regulate gene 3. The feedforward motif appears far more often than chance in natural genomes [48],[49], and presents a variety of temporal information processing possibilities [50],[51]. Among them is the ability to detect sustained, rather than merely transient or accidental, increases in an incoming signal. However, the level of signal that is considered elevated is still preordained.

We consider a more challenging version of the problem in which the statistics of the signal are not known ahead of time. This may be because the operating environment of the cell is not known *a priori* or because the signal itself is difficult to measure experimentally. Whether or not the signal is elevated at a given moment thus depends on the the signal's mean value and typical fluctuations—properties which may themselves change over time. More formally, if the statistics of *I*(*t*) are relatively constant for some period, then a natural definition of elevated is *I*(*t*)>μ*_I_*+*c*σ*_I_*, where μ*_I_* is the mean of *I*(*t*) over time, σ*_I_* is the standard deviation, and *c* is a constant specifying how large an elevation is of interest. To solve the problem, the cell must identify the signal statistics μ*_I_* and σ*_I_*, and then compute whether the signal is elevated at any particular time.

**Figure 5:**
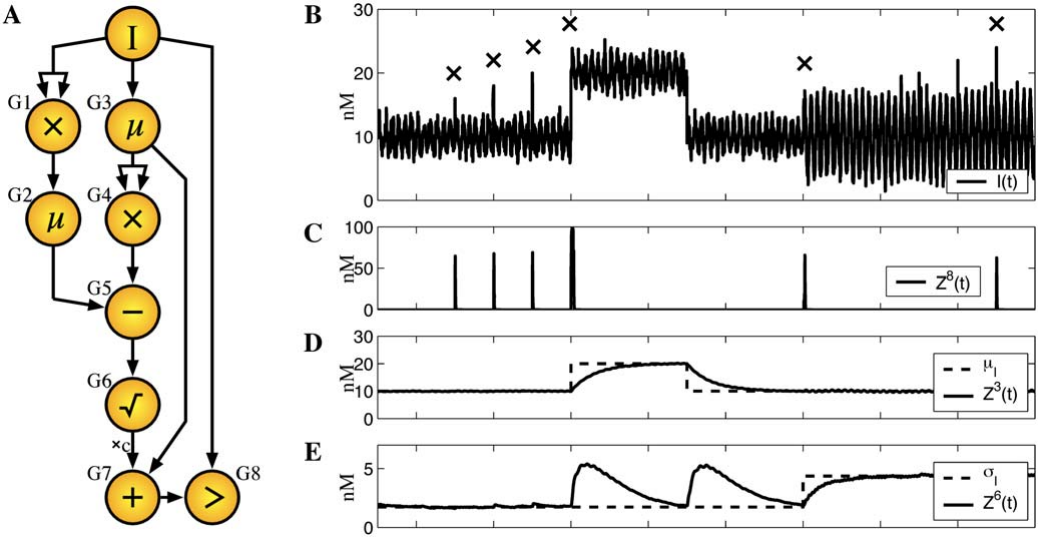
(A) Diagram of a network of arithmetic genes that computes the mean and standard deviation of a time-varying signal, *I*(*t*), and responds when the signal is statistically significantly elevated. Circles represent genes. An arrow between genes G*i* and G*j* means that G*i*'s protein is an input to (transcription factor for) G*j*. Symbols inside the circles denote the operation computed. μ denotes a gene that is activated proportional to its input, but operates at a slower time scale than the other genes, resulting in a recency-weighted temporal average of its input. (B–E) Simulation results. (B) The input signal is primarily a sinusoidal oscillation with Gaussian noise added. The mean changes on days 8 and 11, and the amplitude changes on day 14. There are short spikes in the signal on days 5, 6, 7, 17, 18 and 19. See Materials and Methods for details. An “X” marks each time the signal is significantly elevated compared to its recent mean and standard deviation. (C) The overall response of the network is given by the expression level of gene G8. It correctly flags each significant elevation of the signal and does not respond at any other time. The responses to the input spikes do not last long because the spikes themselves do not last long. The responses to the changes in the oscillations on days 8 and 14 are short because the network quickly adjusts to the changed statistics of the input signal. (D,E) The mean and standard deviation of the sinusoidal oscillations, and the network's recency-weighted estimates of the mean and standard deviation of the signal, as encoded by the concentrations of the proteins for genes G3 and G6.

Figure 5A depicts a network of arithmetic genes that accomplishes this task. The circles represent genes and an arrow from one gene to another means that the first gene's protein acts as an input to the second gene. The network comprises arithmetic genes for multiplication, addition, subtraction, taking the square root, and comparison, as well as two genes labeled by μ. The arithmetic and comparison genes are assumed to reach steady state at a faster time scale than variations in the input signal *I*(*t*). The two μ genes are simply activated proportional to their input and are assumed to reach steady state at a slower time scale than variations in the input signal. Thus, the μ genes effectively compute a recency-weighted time-average of their inputs, the exact nature of which depends on the details of the kinetic parameters. Gene G1 multiplies *I*(*t*) by itself, and because G1 operates at a faster time scale than *I*, we can approximate the expression of its protein as *Z* ^1^(*t*) = *I*(*t*)^2^. Gene G2 averages *Z* ^1^(*t*) over time, so we can approximate its expression as *Z* ^2^(*t*) = *E_t_*(*Z* ^1^(*t*)) = *E_t_*(*I*(*t*)^2^), where *E_t_* denotes averaging over time. By similar reasoning, the expression of genes G3 and G4 is *Z* ^3^(*t*) = *E_t_*(*I*(*t*)) and *Z* ^4^(*t*) = (*Z* ^3^(*t*))^2^ = (*E_t_*(*I*(*t*)))^2^. Genes G5 and G6 compute 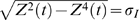. (See Equations 26 and 27 and surrounding discussion for the square root function.) G7 is a modified addition gene which computes *Z* ^7^(*t*) = μ*_I_*+*c*σ*_I_*, for constant *c*, and G8 compares this result to *I*(*t*), turning on when the latter is greater than the former.

To test this network, we simulated a differential equation model of transcript and protein dynamics subject to a time-varying input (see Figure 5B and Materials and Methods). The simulation covered 20 days during which the signal's mean or standard deviation changed three times. There were also six brief spikes in the signal. We chose *c* = 3, so that G8(t) should turn on, to an expression level of 100 nM, whenever *I*(*t*) exceeded its mean plus three standard deviations. Details of the simulation, including the exact equations and kinetic parameters, can be found in the Materials and Methods section.

Figure 5C–E shows the results of our simulation. The simulation covers 20 days of simulated time, though we do not show the first 3 days during which a transient due to the initial conditions disappears. Figure 5B shows the input signal, and Figure 5C shows the overall response of the network to that signal. The network responds at the times that it should and at no other times. Its response to the input spikes and the increase in oscillation amplitude on day 14 are brief and do not reach the fully-on level of 100 nM, mainly because the elevations themselves are brief. When the signal jumps higher on day 8 and remains there, the network responds for longer and reaches a fully-on level. However, this response too vanishes as the network adjusts to the change in signal statistics. (The problem statement requires adjustment to changing signal statistics, so that elevation is relative to the recent mean and standard deviation of the signal. If a more sustained response is desired, G8 could be augmented with a positive feedback loop to keep it activated once triggered.) Figure 5D,E show the mean and standard deviation of the signal's oscillations and the network's recency-weighted estimates of signal mean and standard deviation. Interestingly, changes to the signal mean on days 8 and 11 are initially interpreted by the network as changes in the standard deviation of the signal. Only over the course of several days is the mistake rectified, with the mean estimate shifting to the correct value and the standard deviation estimate returning to its previous, correct level.

### Approximating analytic functions

The elementary arithmetic operations provide a basis for analog computation, in that these operations can be combined to compute more complex functions. However, from the standpoint of the cell, it seems desirable to perform computations with as little biomolecular machinery as possible. From the form of Equation 24, it is not clear how other elementary operations, such as exponentiation, logarithms, or trigonometric functions, might be implemented. Whether these particular functions are of useto cells can be debated, but certainly cells need to compute more complicated functions than elementary arithmetic. One way to approximate more complicated operations is by power series expansions. The focus of this section is on analytic functions—functions with Taylor series expansions that converge pointwise to the correct values, at least for some range of inputs. For simplicity, we consider single-input functions. Generalization to multiple-input functions is straightforward.

**Figure 6:**
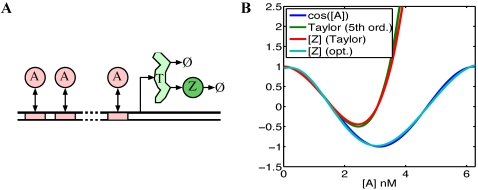
Approximation of analytic function. (A) Schematic of interactions for a gene regulated by a single transcription factor, *A*, via *N* independent binding sites. (B) For varying input levels, [*A*], the four curves represent: cos([*A*]), the 5^th^ order Taylor series approximation of *cosine* centered at zero, the steady state output ([*Z*]) of a pair of genes computing the Taylor series approximation to *cosine*, and the steady state output when kinetic rates are optimized so that [*Z*]≈cos([*A*]), over the range [*A*]∈[0,2π] nM.

Figure 6A shows a schematic of a model describing a single gene regulated by one type of transcription factor, denoted by *A*. We assume *N* binding sites for *A* on the gene's promoter, each acting independently with equilibrium association binding constant *K*. We assume that transcription rate depends only on the number of binding sites occupied by *A*. This model is formalized by the reaction equations below.
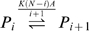
(34)

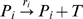
(35)

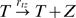
(36)

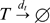
(37)

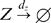
(38)Here, *P_i_* denotes the promoter bound by *i* copies of factor *A*. *T* denotes transcript, and *Z* denotes protein. The steady state expression of protein *Z* is
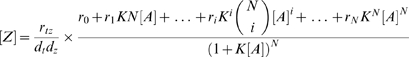
(39)This can also be written as 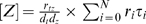, where 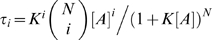 is the fraction of time *i* binding sites for *A* are occupied. Now, consider an analytic function *f*(*x*) with power series expansion
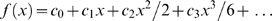
(40)Suppose first that all the *c_i_* are nonnegative. Then the gene can approximate the function up to order *N* by assuming *K*[*A*]≪1, so that the term (1+*K*[*A*])*^N^* is approximately one, and matching the coefficients in Equations (39) and (40).
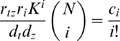
(41)If some *c_i_* are negative, then the series can be divided into positive and negative components.
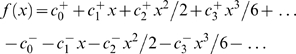
(42)where *c_i_* ^+^ = max(*c_i_*,0) and *c_i_* ^−^ = −min(*c_i_*,0). Two genes of the type in Figure 6A can then combine to compute the function, one computing the positive part of the series and one computing the negative part. The difference in the protein concentrations of the two genes approximates *f*.

For example, suppose the cell wanted to approximate the cosine function using a pair of genes of the type in Figure 6A with five binding sites. The Taylor series expansion for cosine, centered at zero, has coefficients *c*_0_ = 1, *c*_1_ = 0, *c*_2_ = −1, *c*_3_ = 0, *c*_4_ = 1, … The blue and green curves in Figure 6B show, respectively, the cosine function and its 5th order Taylor series approximation. Using biologically plausible parameters for the transcription factor binding affinity, translation rate, and rates of transcript and protein decay (see Materials and Methods), Equation 41 can be solved for the necessary transcription rates, *r_i_*. The red curve in Figure 6B shows [*Z*] = [*Z* ^+^]−[*Z* ^−^], where [*Z* ^+^] is the steady state protein concentration for the gene approximating the positive portion of the series, and [*Z* ^−^] is the steady state protein concentration for the gene approximating the negative portion of the series. The Taylor series approximation and [*Z*] match each other closely, though neither is a good approximation to cos([*A*]) above a concentration of about [*A*] = 2 nM. If the cell places importance on approximating the function at higher concentrations, then different parameters are need. The cyan curve in Figure 6B shows shows [*Z*] for the same two-gene system when parameters are optimized to minimize the mean square error between [*Z*] and cos([*A*]) over the range [*A*]∈[0,2π] Nm. (See Materials and Methods for details.) Sacrificing quality only slightly near zero allows the gene pair to capture a full period of the function, while retaining biologically plausible parameters.

## Discussion

We have shown that different parameterizations of a simple model of transcriptional regulation can reproduce binary arithmetic operations (addition, zero-truncated subtraction, multiplication, division), various inequality comparisons, and fractional powers. Unary, ternary, quaternary, etc. operations can be similarly implemented at a biochemical level. These models are not the only way, and may not be the best way, that these operations can be implemented. For example, we have not considered models with cooperativity between binding sites, allosteric features for transcription factors, regulation of translation or degradation, or chromatin-related mechanisms. One way to explore the utility of these mechanisms would be to perform an explicit computational search through some appropriate space of allowed reaction systems (as in Deckard and Sauro [34], for example), searching for systems with superior performance in terms of various metrics (such as accuracy, noise, robustness, evolvability, or energy consumption). Nevertheless, our simple models demonstrate the possibility of arithmetic computations at the level of transcriptional regulation.

Other functions, such as exponentiation, logarithms, and trigonometric functions, could be computed by combinations of the arithmetic operations. However, this would be energetically wasteful and would require many genes. A more efficient approach is toapproximate directly by using more complicated regulatory machinery. We showed that a pair of genes, each with *N* binding sites for a transcription factor, can approximate any analytic function up to order *N* by reproducing its power series approximation. We also showed that by adjusting the parameters of such a gene pair, the analytic function can be approximated well over a larger range of inputs. While this drastically reduces the number of genes used to compute such functions, other mechanisms may be yet more efficient, and this is an avenue for future research.

Our emphasis on arithmetic computations stands in contrast to work on logical or Boolean notions of computation in genetic networks. Interestingly, Hjelmfelt *et al.* [12] and Magnasco [13] both considered the problem of addition, but in the binary sense, with different chemical species effecting each bit of the operation. In general, there is no relationship between an arithmetic interpretation of a gene's behavior—which is accurate for a limited range of transcriptionfactor concentrations—and a logical interpretation—which focuses on the extremes of low and high transcription factor concentrations. For example, in our model for an Addition gene, transcription occurs whenever factor A or factor B is bound, keeping in mind that factors A and B cannot bind simultaneously (Figure 2A). Such a gene could be interpreted as implementing the logical OR function, because it would be expressed if either factor A or factor B is in high concentration. Recall, however, that an alternate design for an Addition gene allows A and B to bind simultaneously, as long as there was no transcription when both were bound. This gene would be interpreted as implementing XOR, as it would be expressed if A or B, but not both, were in high concentrations. Conversely, our designs for the zero-truncated subtraction function and the greater than function have identical logical structure (Figure 2A), but behave quite differently as arithmetic operations. Thus, a gene's arithmetic behavior, if any, is not determined by its logical behavior and vice-versa. This is an important caveat for the interpretation of empirical measurements of gene response surfaces, which may be probed at non-physiological concentrations of regulatory molecules.

For our gene models to accurately approximate the desired operations, certain products of kinetic parameters must be equal. For example, the multiplication gene requires that *r_tz_r_ab_K_oa_K_ab_* = *d_t_d_z_*. Such a constraint may be biologically implausible or difficult to maintain over different cellular conditions. If the constraint did not hold, the expression of the multiplication gene would still be proportional to the product of the transcription factor expression levels—the role of the constraint is only to scale the output expression correctly. Thus, violation of the constraint in a particular situation need not invalidate the interpretation of the gene as performing multiplication. The case is similar for all the genes in Figure 2 except the addition gene. This gene requires *r_tz_r_a_K_oa_* = *r_tz_r_b_K_ob_* = *d_t_d_z_*. If those equalities failed, but *r_a_K_oa_* = *r_b_K_ob_*, then the expression of the gene would still be proportional to the sum of the transcription factor expression levels. If the latter equality failed as well, then the gene could be interpreted as performing a weighted sum. Indeed, we took advantage of this possibility in our design for the network that detects significant elevations in a time varying signal (see gene G7 in Figure 5A).

In our models, precision (noise) in the output depends on the transcription factor binding and unbinding rates, while the accuracy of the output depends only on the ratio of the rates. This implies that there is no trade-off between precision and accuracy. Each can be selected independent of the other, by evolution or by synthetic biologists, subject to biological limitations on the parameters. We have not analyzed the speed with which these networks reach equilibrium, which could also be an important factor in some settings. Indeed, in many processes, such as development, expressing genes at the correct time is just as important as expressing them at the right level. Further, some genetic networks, such as circadian networks [52] or the feed-forward motif [50],[51], exist solely to keep time or process temporal signals. Our design and analysis of a network that tracks the first and second moments of a time varying signal and responds when the signal is statistically significantly elevated demonstrates that genes performing arithmetic computations can be useful in temporal information processing. An important avenue for further research is to consider more dynamical notions of computation, including investigation of how biochemical networks might implement computations from sequential signal processing or control theory.

We expect that notions of computation more sophisticated than simple logical operations, already being documented experimentally [4],[5],[32],[53], will play an increasing role in our understanding of transcriptional regulation and of biochemical systems more generally. Our analysis of the analog computational abilities of transcriptional regulatory networks is a step in this direction.

## Materials and Methods

Our Gillespie simulations for the analysis of noise for the Addition gene used a slightly simplified set of equations. Because factors *A* and *B* cannot bind the promoter simultaneously, we lumped the two bound states into one.
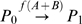
(43)

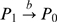
(44)where *P*_1_ denotes the promoter bound by either *A* or *B*, *f* is the forward binding rate of *A* and *B* to the promoter, and *b* is the unbinding rate. Transcription then follows the reaction
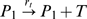
(45)Translation, degradation of transcripts, and degradation of proteins remained the same, following Equations (10) to (12). We used the parameters [*A_tot_*] = [*A*] = 30 nM, [*B_tot_*] = [*B*] = 70 nM, 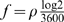 nM^−1^·sec^−1^, b = ρ sec^−1^, *r_t_* = 1 nM·sec^−1^, 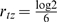 sec^−1^, 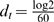 sec^−1^, 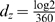 sec^−1^, where ρ is the dimensionless parameter we used to control the binding and unbinding rates of *A* and *B* to the promoter, while leaving the equilibrium association constant unchanged. For different values of ρ, the system was simulated from the initial condition *P*_0_ = 1, *P*_1_ = *T* = *Z* = 0, for one million reactions. Figure 4A plots initial portions of several of those trajectories, but Figure 4B,C are based on the full one million reactions.

Our simulations of the network for detecting elevated signals involved 17 variables: the exogenous input *I*(*t*), and a transcript concentration *T^i^*(*t*) and protein concentration *Z^i^*(*t*) for each gene *i*. All variables have units nanomolar (nM). The input signal was primarily a sinusoidal oscillation with a period of four hours. Oscillations had mean 10 and amplitude 2 for the first eight of the 20 days simulated, mean 20 nM and amplitude 2 nM for the next three days, mean 10 nM and amplitude 2 nM for another 3 days, and mean 10 nM and amplitude 6 nM for the final six days. An independent Gaussian disturbance with mean zero and variance one was added to the signal during every 10 minute period. At the starts of days 5, 6, 7, 17, 18 and 19, there were 10 minute spikes in the signal at levels 16, 18, 20, 20, 22 and 24 nM respectively. We assumed the transcription factor binding equations were at steady state in order to speed the calculations. Transcription was driven at the rate expected based on the transcription factor concentrations. The exact differential equations simulated were:
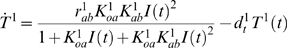
(46)

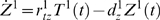
(47)

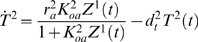
(48)

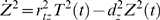
(49)

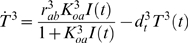
(50)

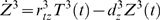
(51)

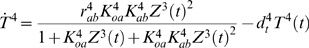
(52)

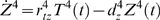
(53)

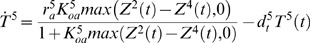
(54)

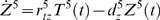
(55)

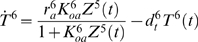
(56)

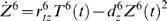
(57)

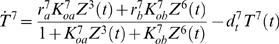
(58)

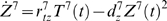
(59)

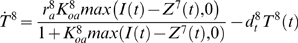
(60)

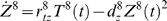
(61)


The system of differential equations was simulated using the ode45 function of MATLAB. Equations 46 through 61 employ several simplifications compared to the equations one would obtain from a direct translation of the kinetic equations in Figure 1, mainly for the purpose of improving numerical stability. For example, we do not explicitly model the heterodimer of *Z* ^2^ and *Z* ^4^ called for by gene 5, which subtracts one concentration from the other. Rather, we drive gene 5 transcription at a rate corresponding to what would be observed if any available *Z* ^2^ and *Z* ^4^ dimerized instantly and irreversibly. Similarly, we do not model production and subsequent decay of the homodimer of *Z* ^6^ suggested by our design for the square root operation. Rather, we simply assume that *Z* ^6^ dimerizes at rate *d_z_* ^6^ and instantly decays.

The kinetic parameters are summarized in Table 4. The “fast” genes were given protein half-lives of 5 minutes and the “slow” genes were given protein half lives of 10 hours. These values are somewhat extreme, but the short half-life is consistent with measurements for actively degraded proteins [54] and both half-lives are within the range of recent measurements in a genome-wide study of yeast [55]. All other parameters are in typical ranges.

**Table 4:** Parameters for the simulation of the network for detected significant elevations in a time-varying signal.

Gene	*d_t_* sec^−1^	*d_z_* sec^−1^	*r_tz_* sec^−1^	*r_a_* nM·sec^−1^	*r_b_* nM·sec^−1^	*r_ab_* nM·sec^−1^	*K_oa_* nM^−1^	*K_ob_* nM^−1^	*K_ab_* nM^−1^
1	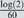	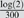	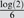			10	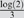		1
2	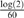	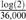	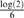	1			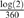		
3	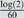	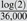	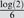	1			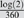		
4	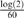	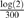	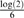			10	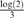		1
5	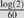	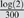	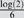	10			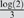		
6	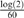	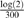	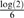	10			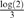		
7	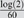	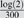	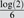	10	10		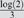	log(2)	
8	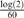	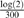	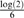	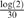			10		

For our study of the cosine approximation, we used parameters 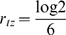 sec^−1^, 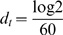 sec^−1^, 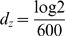 sec^−1^, and *K* = 0.01 nM^−1^. For the direct Taylor series approximation, Equation 41 implied transcription rates *r*_0_^+^ = 5.78×10^−5^ nM·sec^−1^, *r*_2_^−^ = 2.89×10^−2^ nM·sec^−1^, and *r*_4_^+^ = 48.1 nM·sec^−1^. For the optimized curve, we allowed all the parameters to vary, including the transcription rates of the positive and negative genes, and their rates of translation and transcript and protein decay. We use the matlab function “fminsearch” to optimize the parameters, minimizing the mean squared error between steady state [*Z*] and cos([*A*]). Because all parameters should be non-negative, they were reformulated as *p_i_* = exp(*b_i_*) where *p_i_* is the *i^th^* parameter and *b_i_* is the unconstrained parameter that we actually optimized. This resulted in the constrained parameters shown in Table 5.

**Table 5:** Optimized parameters for a pair of genes approximating the cosine function.

Gene	*r_tz_*	*d_t_*	*d_z_*	*K*	*r*_0_	*r*_1_	*r*_2_	*r*_3_	*r*_4_	*r*_5_
+	0.1049	0.0761	0.0013	0.0972	0.0020	0.0018	0	0	1.1420	0.0003
−	0.1471	0.0031	0.0007	0.0419	0	0	0	0.0161	0.0214	0

Units for the parameters are the same as in Table 4.

## References

[1] Arkin AP, Walleczek J (2000). Signal processing in biochemical reaction networks.. Self-Organized Biological Dynamics and Nonlinear Control.

[2] Regev A, Shapiro E (2002). Cells as computation.. Nature.

[3] Guet CC, Elowitz MB, Hsing W, Leibler S (2002). Combinatorial synthesis of genetic networks.. Science.

[4] Setty Y, Mayo AE, Surette MG, Alon U (2003). Detailed map of a cis-regulatory input function.. Proc Natl Acad Sci U S A.

[5] Mayo AE, Setty Y, Shavit S, Zaslaver A, Alon U (2006). Plasticity of the cis-regulatory input function of a gene.. PLoS Biol.

[6] Alberts B, Johnson A, Lewis J, Raff M, Roberts K (2002). Molecular biology of the cell, fourth edition.

[7] Glass L, Kauffman SA (1973). The logical analysis of continuous, non-linear biochemical control networks.. J Theor Biol.

[8] Hjelmfelt A, Weinberger ED, Ross J (1991). Chemical implementation of neural networks and Turing machines.. Proc Natl Acad Sci U S A.

[9] Arkin A, Ross J (1994). Computational functions in biochemical reaction networks.. Biophys J.

[10] Buchler NE, Gerland U, Hwa T (2003). On schemes of combinatorial transcription logic.. Proc Natl Acad Sci U S A.

[11] Sauro HM (2004). The computational versatility of proteomic signaling networks.. Current Proteomics.

[12] Hjelmfelt A, Weinberger ED, Ross J (1992). Chemical implementation of finite-state machines.. Proc Natl Acad Sci U S A.

[13] Magnasco MO (1997). Chemical kinetics is Turing universal.. Phys Rev Lett.

[14] Ben-Hur A, Siegelmann HT (2004). Computation in gene networks.. Chaos.

[15] Bodnar J (1997). Programming the *Drosophila* embryo.. J Theor Biol.

[16] Sanchez L, van Helden J, Thieffry D (1997). Establishment of the dorso-ventral pattern during embryonic development of *Drosophila melanogaster*: a logical analysis.. J Theor Biol.

[17] Mendoza L, Alvarez-Buylla ER (1998). Dynamics of the genetic regulatory network for *Arabidopsis thaliana* flower morphogenesis.. J Theor Biol.

[18] Mendoza L, Thieffry D, Alvarez-Buylla ER (1999). Genetic control of flower morphogenesis in arabidopsis thaliana: a logical analysis.. Bioinformatics.

[19] Sanchez L, Thieffry D (2001). A logical analysis of the gap gene system.. J Theor Biol.

[20] Albert R, Othmer HG (2003). The topology of the regulatory interactions predicts the expression pattern of the segment polarity genes in *Drosophila melanogaster*.. J Theor Biol.

[21] Sanchez L, Thieffry D (2003). Segmenting the fly embryo: a logical analysis of the *pair-rule* cross-regulatory module.. J Theor Biol.

[22] Faure A, Naldi A, Chaouiya C, Thieffry D (2006). Dynamical analysis of a generic boolean model for the control of the mammalian cell cycle.. Bioinformatics.

[23] Gonzalez A, Chaouiya C, Thieffry D (2006). Dynamical analysis of the regulatory network defining the dorsal-ventral boundary of the drosophila wing imaginal disc.. Genetics.

[24] Wolpert L, Waddington CH (1968). The french flag problem.. Towards a Theoretical Biology.

[25] Glass L (1975). Combinatorial and topological methods in nonlinear chemical kinetics.. J Chem Phys.

[26] Ghosh R, Tomlin CJ, Benedetto MDD, Sangiovanni-Vincentelli A (2001). Lateral inhibition through delta-noth signaling: A piecewise affine hybrid model.. Lectures Notes in Computer Science 2034.

[27] de Jong H, Geiselmann J, Batt G, Hernandez C, Page M (2004). Qualitative simulation of the initiation of sporulation in *Bacillus subtilis*.. Bull Math Biol.

[28] Ropers D, de Jong H, Page M, Schneider D, Geiselmann J (2006). Qualitative simulation of the carbon starvation response in *Escherichia coli*.. BioSystems.

[29] Perkins TJ, Jaeger J, Reinitz J, Glass L (2006). Reverse engineering the gap gene network of *Drosophila melanogaster*.. PLoS Comput Biol.

[30] Gardner TS, Cantor CR, Collins JJ (2000). Construction of a genetic toggle switch in *Escherichia coli*.. Nature.

[31] Elowitz MB, Leibler S (2000). A synthetic oscillatory network of transcriptional regulators.. Nature.

[32] Yuh CH, Bolouri H, Davidson EH (1998). Genomic cis-regulatory logic: experimental and computational analysis of a sea urchin gene.. Science.

[33] Hjelmfelt A, Ross J (1992). Chemical implementation and thermodynamics of collective neural networks.. Proc Natl Acad Sci U S A.

[34] Deckard A, Sauro HM (2004). Preliminary studies on the in silico evolution of biochemical networks.. Chembiochem.

[35] Barkai N, Leibler S (1997). Robustness in simple biochemical networks.. Nature.

[36] Alon U, Surette MG, Barkai N, Leibler S (1999). Robustness in bacterial chemotaxis.. Nature.

[37] Rao CV, Wolf DM, Arkin AP (2002). Control, exploitation and tolerance of intracellular noise.. Nature.

[38] Houchmandzadeh B, Wieschaus E, Leibler S (2002). Establishment of developmental precision and proportions in the early *Drosophila* embryo.. Nature.

[39] Eldar A, Dorfman R, Weiss D, Ashe H, Shilo BZ (2002). Robustness of the BMP morphogen gradient in *Drosophila* embryonic patterning.. Nature.

[40] Von Dassow G, Meir E, Munro EM, Odell GM (2000). The segment polarity network is a robust developmental module.. Nature.

[41] Samoilov M, Arkin A, Ross J (2002). Signal processing by simple chemical systems.. J Phys Chem A.

[42] Tkacik G, C GC, Bialek W (2007). Information flow and optimization in transcriptional control.. ArXiv:.

[43] Libby E, Perkins TJ, Swain PS (2007). Noisy information processing through transcriptional regulation.. Proc Natl Acad Sci U S A.

[44] Thattai M, van Oudenaarden A (2001). Intrinsic noise in gene regulatory networks.. Proc Natl Acad Sci U S A.

[45] Gillespie DT (1977). Exact stochastic simulation of coupled chemical reactions.. J Phys Chem.

[46] Swain PS, Elowitz MB, Siggia ED (2002). Intrinsic and extrinsic contributions to stochasticity in gene expression.. Proc Natl Acad Sci U S A.

[47] Alon U (2007). Network motifs: theory and experimental approaches.. Nat Rev Genet.

[48] Lee TI, Rinaldi NJ, Robert F, Odom DT, Bar-Joseph Z (2002). Transcriptional regulatory networks in *Saccharomyces cerevisiae*.. Science.

[49] Shen-Orr S, Milo R, Mangan S, Alon U (2002). Network motifs in the transcriptional regulation network of *Escherichia coli*.. Nat Genet.

[50] Mangan S, Alon U (2003). Structure and function of the feed-forward loop network motif.. Proc Natl Acad Sci U S A.

[51] Mangan S, Itzkovitz S, Zaslaver A, Alon U (2006). The incoherent feed-forward loop accelerates the response-time of the gal system of *Escherichia coli*.. J Mol Biol.

[52] Ko CH, Takahashi JS (2006). Molecular components of the mammalian circadian clock.. Hum Mol Genet.

[53] Andrews BW, Yi TM, Iglesias PA (2006). Optimal noise filtering in the chemotactic response of *Escherichia coli*.. PLoS Comput Biol.

[54] Bachmair A, Finley D, Varshavsky A (1986). In vivo half-life of a protein is a function of its amino-terminal residue.. Science.

[55] Belle A, Tanay A, Bitincka L, Shamir R, O'Shea EK (2006). Quantification of protein half-lives in the budding yeast proteome.. Proc Natl Acad Sci U S A.

